# Evaluation the effect of *Silybum marianum* ointment on episiotomy wound healing and pain intensity in primiparous women: a randomized triple blind clinical trial

**DOI:** 10.1186/s12906-021-03413-z

**Published:** 2021-10-07

**Authors:** Elmira Toomari, Sepideh Hajian, Faraz Mojab, Tayebe Omidkhah, Malihe Nasiri

**Affiliations:** 1 Student Research Committee, School of Nursing & Midwifery, Shahid Beheshti University of Medical Sciences, Tehran, Iran; 2 Department of Midwifery & Reproductive Health, School of Nursing & Midwifery, Shahid Beheshti University of Medical Sciences, Vali Asr Ave., Ayatollah Rafsanjani Cross Road, Niayesh Complex, Tehran, 1985717443 Iran; 3 Midwifery & Reproductive Health Research Center, School of Nursing & Midwifery, Shahid Beheshti University of Medical Sciences, Tehran, Iran; 4 Department of Pharmacognosy, School of Pharmacy, Shahid Beheshti University of Medical Sciences, Vali Asr Ave., Ayatollah Rafsanjani Cross Road, Niayesh Complex, Tehran, 1991953381 Iran; 5 Guilan University of medical science, Shahid Noorani Hospital, Talesh, Guilan Province 4271937916 Iran; 6 Department of Basic sciences, Faculty of Nursing and Midwifery, Shahid Beheshti University of Medical Sciences, Tehran, IR Iran

**Keywords:** Episiotomy, Pain, Wound healing, *Silybum marianum*

## Abstract

**Background:**

Episiotomy is the most commonn surgical procedure in midwifery which as any other wounds can cause infection or delay in healing.

The current study aimed to determine effect of *Silybum marianum* ointment on pain severity and healing of episiotomy wound in primiparous women referring to Shahid Nourani Hospital at 2019.

**Methods:**

This research was done as a randomized, triple-blind clinical trial on 87 priiparous women (44 indivdiuals in *Silybum marianum* ointment group and 43 indivdiuals in placebo group) referred to Shahid Nourani Hospital in Talesh (Guilan Province), Iran at September 2019.

After labor and performing episiotomy, twice a day for 10 days as a fingertip size of the ointment was prescribed to be topically used on the episiotomy incision for both groups (*Silybum marianum* ointment or placebo ointment).

Data gathering was done using demographic and midwifery information questionnaire, Episiotomy healing assessment: Redness, Edema, Ecchymosis, Discharge, Approximation)REEDA Scale (REEDA Scale: Redness(R); Edema (E), Ecchymosis(E), Discharge from the wound(D); Approximation of the perineal tissues(A))(scale, and visual analogue scale of pain. Examination of healing status of the perinea incision was performed during first 12 h, fifth day and tenth day after labor.Kolmogrov-Smirnov test was used in order to investiagte nomrality of data distribution of quantitative data, and two- independent samples t test, Chi square, repeated measures two factorial analysis of variance and Fischer’s exact test were used. SPSS software version 23 was used to analyze data and 0.05 was considered as signifcance level.

**Results:**

Both groups of *Silybum marianum* and placebo groups did not differ regarding demographic and midwifery characteristics, hygiene status prior to intervention (*P* > 0.05).

Mean difference of pain severity and REEDA scale in *Silybum marianum* ointment group in 12 h after labor, at fifth day and tenth day after labor was significant comparing to control group which indicates decline in episiotomy pain severity and faster wound healing (*P* < 0.001).

**Conclusions:**

*Silybum marianum ointment* ointment accelerates episiotomy wound healing rate due to its healing properties and decreases pain severity.

**Trial registration:**

This study was registered in Iranian Registry of Clinical Trials in 10/08/2019 with the IRCT ID: IRCT201811100411603N1.

**Supplementary Information:**

The online version contains supplementary material available at 10.1186/s12906-021-03413-z.

## Background

Episiotomy means pudendal incision. Perineotomy refers to perinea incision. This incision is made at the second phase of labor in order to increase size of soft tissue space of the pelvic outlet, prevent ruptures, facilitate labor, and decrease fetus labor time and is done as two forms of medial and lateral incision [1].

Recommendation on undergoing episiotomy is indicated in the conditions including: breech presentation, labor by forceps or vacuum, permanent occiput posterior, and cases in which not performing episiotomy might cause perinea rupture and need to premature labor due to worry on fetal heart pulse [1, 2].

There is considerable increase in episiotomy worldwide [3]. More than 85% of the labors in Vietnam are done through vaginal method which amongst them, 100% of primiparous women underwent episiotomy [4]. In addition, prevalence of episiotomy is reported as 47.8% [5] in Erbil, Iraq, 56.3% in Turkey [6], 63.1% in Enugu, western south of Nigeria [7], 8% in Netherlands, 20% in England, 28% in Argentina, 40.6% in Austria, 50% in United States, and 54% in Northern America [8].

There is no comprehensive report on performing this surgical incision in Iran, but in most hospitals of the country, it is considered as common method specifically for birth of neonates of primiparous women [9]. However, as any other incisions, some complications are reported for this procedure including bleeding, increase of incision toward anal sphincter, perinea pain, dyspareunia, infection, perinea hematoma, and formation of recto-vaginal fistula [2], and due to involvement of pelvic muscles, it can interfere with many usual activities such as sitting, walking, standing, huddling, urination, and defecation, and disturb the mother who already delivered [10]. Results of the studies showed that perinea damage does not only cause physical damage but also induce emotional and mental damages [11].

Numerous factors are effective in healing of wounds, such as, upon local wound factors, systemic mediators, the injury type [12], infection, hypotension, age, nutritional status, systemic diseases, exposing to radiation, estrogen level, medication use, mental and psychological status, age, wound type [13], smoking, pulmonary diseases and peripheral vascular disease (PVD) result in hypoxia [14], wound leading cause, Ideal Dressing [15], duration of diagnosis of the wound until surgery, factors related to efficacy of treatment of wound surgeries [16].

Episiotomy incision is healed during 3 weeks after labor naturally and without any confounding factors such as infection [1]. Phases of wound healing include as, hemostasis/inflammation, proliferation, and remodeling [17]. However, edema, redness, bruising are reported in 26.2, 6.6, 3.3% of women, respectively who underwent episiotomy in first 24 h after labor [18]. Perinea pain severity can go on in first day beyond 90% and in 88% of cases; this pain is continued over 2 months after labor [19].

Numerous attempts can be done after labor following episiotomy to reduce perinea pain and accelerate wound healing, which are applicable through two methods of pharmaceutical and non-pharmaceutical methods (cryotherapy [20], laser therapy [21], pelvic floor muscle trainings [22], acupuncture [23], and perinea massage [24], and electrical stimulating [25]). There is increasing tendency in the world specifically in Iran in recent years to investigate the physiologic and pharmacologic effects of these herbal compounds and using them which the most important reasons of the tendency to these approaches include: lesser side effects, variety of the effective compounds in the herbs, lower specific costs comparing to chemical pharmaceutical elements, development of industries depended to culturing medicinal herbs, prevention from currency outflow from the country, producing useful job [26]. Despite having a few evidence of the above mentioned herbal agents efficacy on episiotomy recovery, there are no formal recommendations for wound healing after episiotomy in Iran including use of herbal products and medical advice for episiotomy care are summarized in adherence to personal hygiene and patient self-care basics such as using regular warm bath and pain killers but herbal remedies are not recommended by physicians routinely owing to the lack of national recommendations in this field.

Amongst medicinal herbs compounds such *Hypericum perforatum* cream [27], chamomile cream [11], *Aloe Vera* Ointment [28], *Ananas comosus* [29], *Curcuma Longa* Ointment [30], *Aloe vera* and *Calendula persica* Ointment [31], one of the herbal compounds recommended for its properties including wound healing in the studies is *Silybum marianum* (*S. marianum)* [32]*. S. marianum* is a plant belonged to family Asteraceae which is also named as Khar Maryam, Maritighal, in Persian languages [33]. This annual and biennal plant grows to a height of 1.5 m and is wildflower [34]. *Silybum marianum* grows in European, Asian, and American countries. Disturbtion of this plant in Iran, is in areas of Gonbad Kavoos, Gorgan, Kelardasht, Mughan desert, Posht kooh, Ahvaz, Shoush and Kazerun [35].

The most important reasons which can be pointed in order to use this herb are the properties of anti-inflammatory, stimulating collagen synthesis, angiogenesis, vasodilatation, decline in bleeding and edema of the wound which are done in microscopic studies [8].

*S. marianum* contains numerous compounds including: sallying A and B, silydianin, silychristin, Apigenin, dehydrosilyn, deoxysilychristin and deoxydianin. *S. marianum* is confirmed to be effective in treatment of hepatic, renal, dermatological diseases, blood sugar control and lipid metabolism, immune system boosting and anti-carcinogen [36].

Silybin is the main component of silymarin effective in synthesis of collagen type 1 [37]. Silymarin improves wound healing process by increase in stromelysine 1 gene expression and extracellular matrix components including glycosaminoglycans and collagen content [38] and has strong antioxidant power which helps prevention of oxidative damage and progression of treatment process, therefore, effect of silymarin on wound healing process can be attributed to the effect of epithelialization and inflammation reduction [39] due to its antioxidant and anti-inflammatory properties [40].

It has been showed that silymarin ointment is a promise therapeutic agent for wound healing in rats for such properties [41]. In addition, findings of a study on the patients with second-degree fire burns indicated that at least 1 month use of oral silymarin lead to faster injury recovery compared to the placebo [42].

Owing to the antioxidant, antimicrobial, antifungal, anti-inflammatory, and analgesic properties of *S. marianum* in a few studies [8, 43, 44], and due to that there is no research to date through randomized controlled design in order to assess the topical *Silybum marianum* seed ethanol extract on wound healing in human, and since there is a need to use of effective, low risk, accessible and low-cost methods for postpartum women, the current study aimed to determine effect of *Silybum marianum* ointment on wound healing and pain severity of episiotomy in primiparous women.

## Methods

### Study design and participants

This is a randomized, triple-blind clinical trial performed to assess effect of *Silybum marianum* ointment on wound healing and pain severity of episiotomy in primiparous women in 2019. This trial adhered to CONSORT guidelines and included CONSORT checklist as a supplementary file 1.

Sample size was estimated using the article by Taleb et al. [45] and by considering 90% test power and the observed effect size of 0.70 in each group at 42 individuals which was increase to at least 45 individuals in each group by considering attrition rate).)

Incusion criteria were as follow: low risk pregnancy, being primiparous, age range of 18–35 years old. BMI of 18.5–29.9, resident in Talesh, ability of reading and writing, singleton birth with cephalic presentation. Vaginal labor with media-lateral episiotomy, without rupture andwithout strumental delivery, no rupture in corioamnion membrane longer than 24 h, normal and spontaneous placental abruption up to 30 min after neonate abortion, no perineal swelling immediately after episiotomy, lack of history of topical and herbal medications allergy, lack of history of previous damage or surgery or visible lesions in perinea.

Exclusion crietria inlcudes: lack of referral of mother to hospital in fifth an tenth day after labor, lack of tendency to continue partcipating in the study, not using ointment regularly and based on instructions, not using washing serum for washing sutures location regularly and based on the instructions (lesser than twice a day), initiation of sexual intercourse during 10 days after labor, occurrence of allergy or complications related to the medication (which in case of incidence of allergy, the particpant was excluded from the study and reported).

### The study tools

Instruments include: 1-The participants’ characteristics and midwifery information questionnaire, 2-the antibiotic use record checklist, 3- side effects of the medication and health status sheet, 4- perinea improvement assessment checklist, 5- visual analogue scale.

Side effects of the medication include allergy, infection, itching, burning, stinging, dryness in the region of wound, fever and ague and health status sheet including six questions on health issues to assess adherence to perinea health by each participant which was used in two studies previously [27, 46] and was made by the researcher. Score of 0–1 was allocated to each question. Then, based on the scores obtained, health status of the samples was classified in three levels of poor (0 to 2), medium (3 to 4) and good (5 to 6).

Perinea recovery assessemnt checklist: disposable paper ruler was used in order to assess perinea recovery and the assessment was done in lithotomy status and through using examination light by the REEDA scale. This tool conisists of five variables or criteria which investigates oedema, bruising, erythema, wound secretions, and wound edges adherence after episiotomy, which this tool is used to assess recovery of perinea wound in previous studies [47, 48]. A score of 0–3 is allocated to each variable in the scale. Score of each variable is computed seperately, sum of the scores are 0–15, and the closer score to 15 indicates greater traums [49].

Visual analogue scare of pain: this tool was used in order to measure pain severity which is as a 10-cm ruler with 11 numbers. The ruler is numerized from 0 to 10, in which zero shows no pain, 1–3 shows mild pain, 4–7 shows moedrate pain, and 8–10 shows severe pain.

In order to determine reliabiity of the health status assessment sheet, test -retest method was used on ten primiparous women before sampling the reasearch subjects within 2 weeks. Reliability and validity of the perinea recovery assessment form was confirmed in a few studies [50, 51].

Inter rater agreement method was used to reassure the consensus between two examiners (researcher and her colleague who are at the same level regarding job experience and scientific level), as ten samples were selected and their wound healing scores were assessed separately. Then correlation coefficient of the assessment scores between two examiners was computed.

Reliability of the disposable ruler was assessed with a non-stretch tape (Laica, Italy) with accurateness of 1 mm.

Reliability and validity of the visual sclae of pain was confirmed in various studies [52–54].

### Study material

*S. marianum* seeds (Asteraceae) were bought from herbal market in Tehran on September 2019. After confirm the seeds (in Medicinal Plant Lab., School of Pharmacy), they were powdered and extracted through soaking in ethanol (maceration X 4), the extracts were mixed together and were concentrated in fewer of 40 °C, According to the similar studies [27, 55] the ointment with concentration of 3% was prepared from this extract with a eucerin basis, and the 30 g tubes were filled. Placebo was prepared with eucerin and in the similar tubes and was named with the codes of A and B, respectively. Both ointments were autoclaved. Total flavonolignans (as silybin) using ultraviolet spectrophotometry was determined at 1.53%.

In order to blind samples and researchers on the type of medication used in two study groups, intervention and control groups (gold standard group), the ointments were made the same in color, odor, and medication shape.

### Study implementation

Sampling was done through purposive sampling method and based on includion criteria. After explaining of the study objectives to the all participants and obtaining their written consent, they entered the study and assigned into the intervention and control groups (gold standard group) through random allocation method using Excel software, version 2019. First, in a column, groups A, B and below were imported; Because the number of samples in each group was determined, therefore, 45, B, A would enter into the following row. In the other column, Using the command RAND, the random numbers were generated. In the next step, using the Sort order, random numbers generated from small to large or reversed, which caused the order of the groups, A and B, to change. Using the new order, people are assigned to different groups. After that, the software determines how to allocate people based on random numbers. For example, in random allocation, the first person may be allocated to the placebo, the third person would be in the intervention group, and so on.

During the hospitalization in labor, in case of emergency condition of a participant such as cesarean section, she was excluded from the study and another eligible participant was replaced until the samples size was completed.

In order to decrease assessment bias and increase of accuracy of the data, the researcher, partcipants and statistician were not awared of the used medication. The participants’ characteristics and midwifery information forms were completed by the first author at the beginning of the labor admission and other necessary data were recorded after the childbirth.

In order to increase internal validity of the study, the participants were matched regarding confounders such as episiotomy (media-lateral), wound repairing method, type of thread, amount of anesthetic substance before incision and during wound repairing, birth attendant, and Apgar score at first and fifth minute. Furthermore, in order to minimize random error, two colleagues who were the same for educational level and job experience in labor recorded items of the REEDA checklist and control of perinea healing.

In order to blinding in the current research, drug and placebo was encoded by the pharmacist; so that the researcher and samples were not awared of the drug content. In addition, all the assessments were evaluated and recorded by the researcher and her colleague who were not aware of the type of drug.

Required instructions on way of caring of the sutures, adherence to personal hygiene, and washing the sutures with washing serum of normal saline, adherenece to sexual health, nutrition and physical activity level were provided by the researcher.

Two hours after labor, firstly one basic assessment was done on episiotomy wound and the directions of use of the medication was explained to each participant by the researccher. So that each partciapnt should use the ointment thoroughly and as 2 cm on the suture twice daily in 10 days after washing hands and drying the perinea region. According to the usual care of the postpartum ward, 500 mg of cefalexin capsule every 6 to 7 h was prescribed to all the women after labor.

Each partcipant was asked to contact the researcher in case of any problem and complication including allergy, infection, itching, burning, stinging, dryness in the area of wound and ague to perform assessemnt and required attempts including urgent need on referral to clinic of postpartum care. All the partcipants were recommended to refer triage ward of the hospital at fifth and tenth day and after discharge, they were called prior to any visit to remind second and third visits.

### Statistical methods

Primary data was assessed using Kolmogorov-Smirnov test to investigate normal distribution of quantitative data. Then, two independent –samples t- test, chi-square, repeated measure two factorial analysis of variance and Fischer’s exact test were used. In order to analyze data, SPSS software version 23 was used and significant level was considered as less than 0.05.

### Ethical considerations

This study was approved in the Organizing Committee of Ethics in Medical Research at Shahid Beheshti University of Medical Sciences as the Committee of Ethics in Research of the Schools of Pharmacy, Nursing and Midwifery -Shahid Beheshti University of Medical Science on 15/4/2019 by assigning the ethical code of IR.SBMU.PHARMACY.REC.1398.030 and was also registered in IRCT with the number: IRCT201811100411603N1 on 10/8/2019.

Women eligible to participate in the study were enrolled voluntarily and with written consent at the beginning of the study and with a commitment not to impose costs on participants, having the right to dispense as well as confidentiality of information.

## Results

**Fig. 1:**
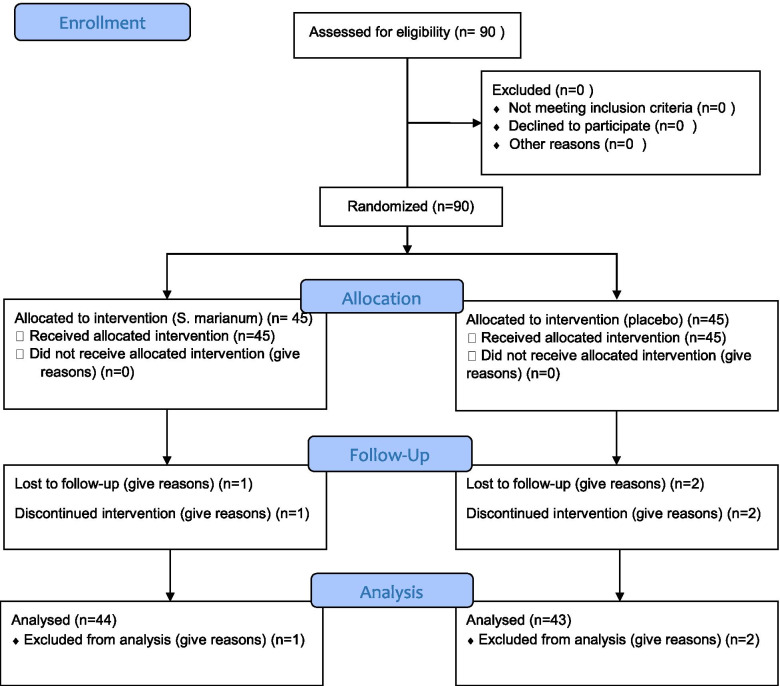
CONSORT flowchart of inclusion and exclusion of the study participants

Out of the 90 participants at the beginning of the study, 43 individuals were in the placebo group and 44 one in the *S. marianum* group. Three subjects (2 in the placebo group and 1 in the *S. marianum* group) were excluded (Fig. 1).

Data analysis showed no significant difference among two study groups on demographic and midwifery characteristics prior to the intervention (*P* > 0.05), scores obtained from health status forms in two groups were computed and compared in order to assess health status of the two groups, which two groups did not differ statistically significant (*P* > 0.05) (Table 1).

**Table 1:** Demographic characteristics of the 2 groups

Treatment Categories			
**Variable**	*S. marianum* **ointment**	**Placebo**	***p*-value**
	Mean/SD	Mean/SD	
Age (years)	26.41 ± 4.77	18.24 ± 5.38	*P = 0.14
BMI(m2Kg)	25.31 ± 1.89	25.78 ± 2.23	*P = 0.28
Birth Weight (gram)	3364.72 ± 281.9	3392.79 ± 79	*P = 0.30
Neonate Head circumference (cm)	38.93 ± 1.5	34.27 ± 1.68	*P = 0.28
Duration of first stage of labor (minute)	146.52 ± 71.02	125.23 ± 1.57	*P = 0.14
Duration of second stage of labor (minute)	17.87 ± 13.94	21.51 ± 19.43	*P = 0.54
Duration of third stage of labor (minute)	4.52 ± 1.57	5.32 ± 2.42	*P = 0.80
Time rupture of membranes (minute)	168.95 ± 120.12	189.53 ± 126.16	*P = 0.43
Number of sutures on skin	0.52 ± 5.09	0.69 ± 5.18	**P = 0.52
Number of analgesics used	25.78 ± 2.23	38.93 ± 1.50	**P = 0.10
**Treatment Categories**			
**Variable ointment**	*S. marianum*	***Placebo***	***p-value***
	Number (%)	Number (%)	
Educational level (woman)			
Elementary	25(11)	16.3(7)	
Middle school	22.7(10)	27.9(12)	**P = 0.78
High school	43.2(19)	46.5(20)	
University	9.1(4)	9.3(4)	
Health status			
absolutely desirable	2(4.5)	1(2.3)	
Approximately desirable	38(86.4)	38(4.88)	**P = 0.85
Undesirable	4(1.9)	4(9.3)	

*Independent t-tests **Mann Whitney

In first 12 h after labor, two groups did not significantly differ regarding none of the REEDA variables (*P* > 0.05) (Table 3). However, the REEDA mean difference score in *S. marianum* ointment group in 12 h after childbirth, and either REEDA mean difference score or all its variables scores at fifth day and tenth day were statically significant comparing to control group which indicates decline in episiotomy wound healing (*P* < 0.001) (Tables 2 and 3).

**Table 2:** Comparison of mean scores of pain severity and wound healing based on assessment time in two groups

**Treatment Categories**			
**Variable** **Pain severity group**	**12 h after labor**	**Fifth day after labor**	**Tenth day after labor**
	**Mean/SD**	**Mean/SD**	**Mean/SD**
*S. marianum* ointment	6 ± 2.03	2.95 ± 2.03	0.11 ± 0.53
Placebo ointment	7.39 ± 2	2.07 ± 5.20	7.39 ± 2
*p*-value		*P < 0.001	
**Treatment Categories**			
**Variable** **Wound healing**	**12 h after labor**	**Fifth day after labor**	**Tenth day after labor**
	**Mean/SD**	**Mean/SD**	**Mean/SD**
*S. marianum* ointment	0.38 ± 0.75	0.27 ± 0.54	0.22 ± 0.52
Placebo ointment	1.02 ± 1.18	1.65 ± 1.54	1.81 ± 1.82
*p*-value		*P < 0.001	

*Two way repeated measure Test for comparing two groups

**Table 3:** Comparison of REEDA Scale in two groups

**Treatment Categories**				
**Variable labor** ***S. marianum*** **ointment**	**Before intervention**	**12 h after labor**	**Fifth day after**	**Tenth day after labor**
	**Mean/SD**	**Mean/SD**	**Mean/SD**	**Mean/SD**
**Redness**	0.25 ± 0.57	0.15 ± 0.42	0.04 ± 0.21	0.04 ± 0.21
**Edema**	0.15 ± 0.36	0.09 ± 0.29	0.02 ± 0.15	0.02 ± 0.15
**Ecchymosis**	0.13 ± 0.40	0.13 ± 0.40	0.04 ± 0.21	0
**Discharge from the wound**	0	0	0	0.15 ± 0.42
**Wound edge continuity**	0	0	0.15 ± 0.36	0
**Treatment Categories**				
**Variable** **Placebo ointment**	**Before intervention**	**12 h after labor**	**Fifth day after labor**	**Tenth day after labor**
	**Mean/SD**	**Mean/SD**	**Mean/SD**	**Mean/SD**
**Redness**	0.32 ± 0.56	0.32 ± 0.56	0.34 ± 0.52	0.30 ± 0.51
**Edema**	0.29 ± 0.46	0.20 ± 0.46	0.20 ± 0.46	0.20 ± 0.46
**Ecchymosis**	0.27 ± 0.63	0.34 ± 0.65	0.32 ± 0.64	0.16 ± 0.43
**Discharge from the wound**	1	0.46 ± 0.21	0.11 ± 0.32	0.86 ± 0.98
**Wound edge continuity**	1	0.46 ± 0.21	0.65 ± 0.75	0.27 ± 0.59
**Variable** *P-value	**Before intervention**	**12 h after labor**	**Fifth day after labor**	**Tenth day after labor**
**Redness**	*P* = 0.32	*P* = 0.25	*P* = 0.003	*P* = 0.003
**Edema**	*P* = 0.59	*P* = 0.34	*P* = 0.04	*P* = 0.04
**Ecchymosis**	*P* = 0.53	*P* = 0.19	*P* = 0.01	*P* = 0.04
**Discharge from the wound**	1	*P* = 0.14	*P* = 0.03	*P* = 0.01
**Wound edge continuity**	1	*P* = 0.14	*P* = 0.001	*P* = 0.001

* Mann Whitney

**Fig. 2:**
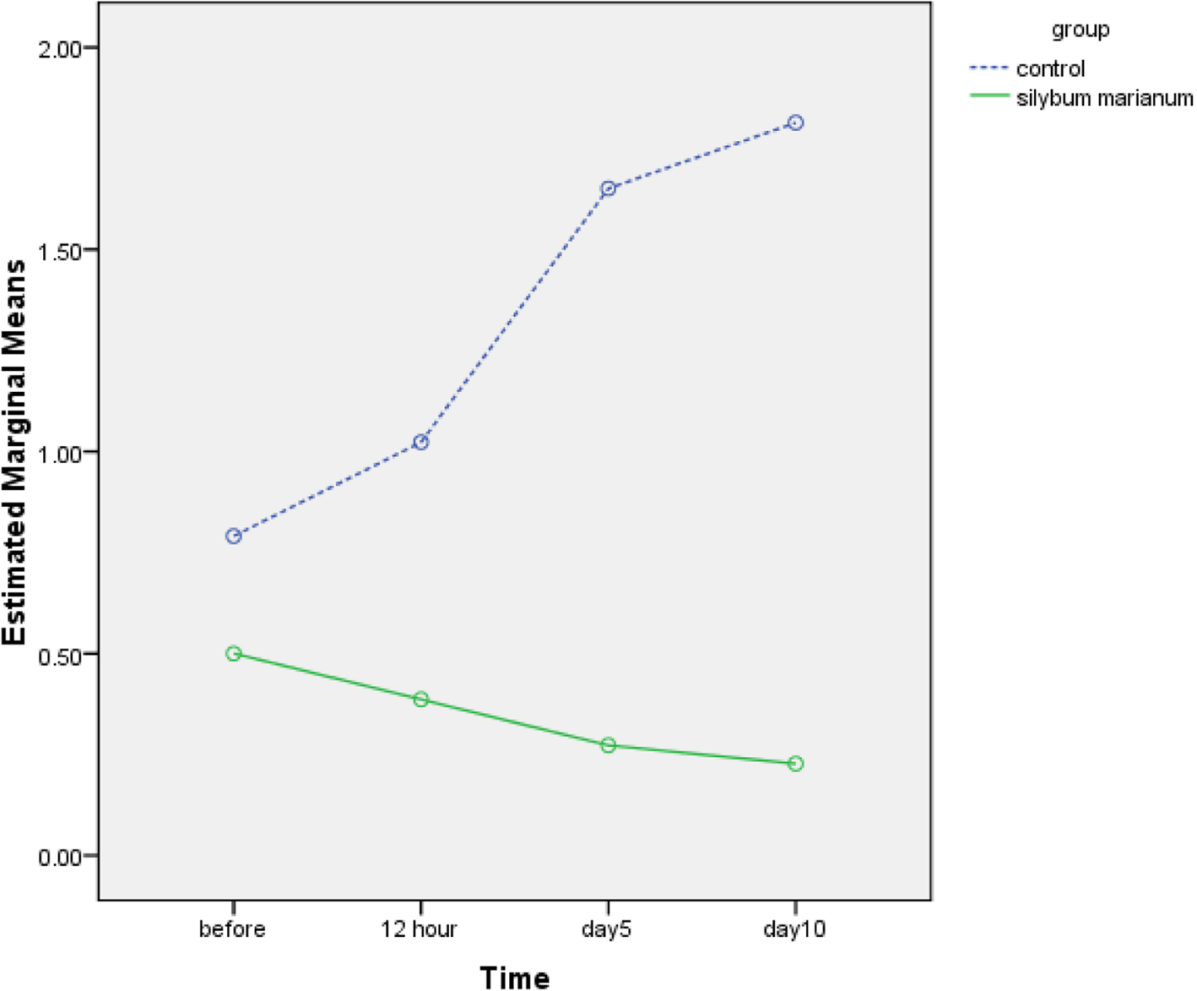
Comparison of healing in two groups of *S. marianum* ointment and Placebo ointment in primiparous women

Figure 2 shows wound healing observed in two groups at different times.

Mean difference of pain severity in *S. marianum* ointment group in 12 h after labor, at fifth day and tenth day was statically significant comparing to control group which indicates decline in episiotomy pain severity (*P* < 0.001) (Table 2).

**Fig 3:**
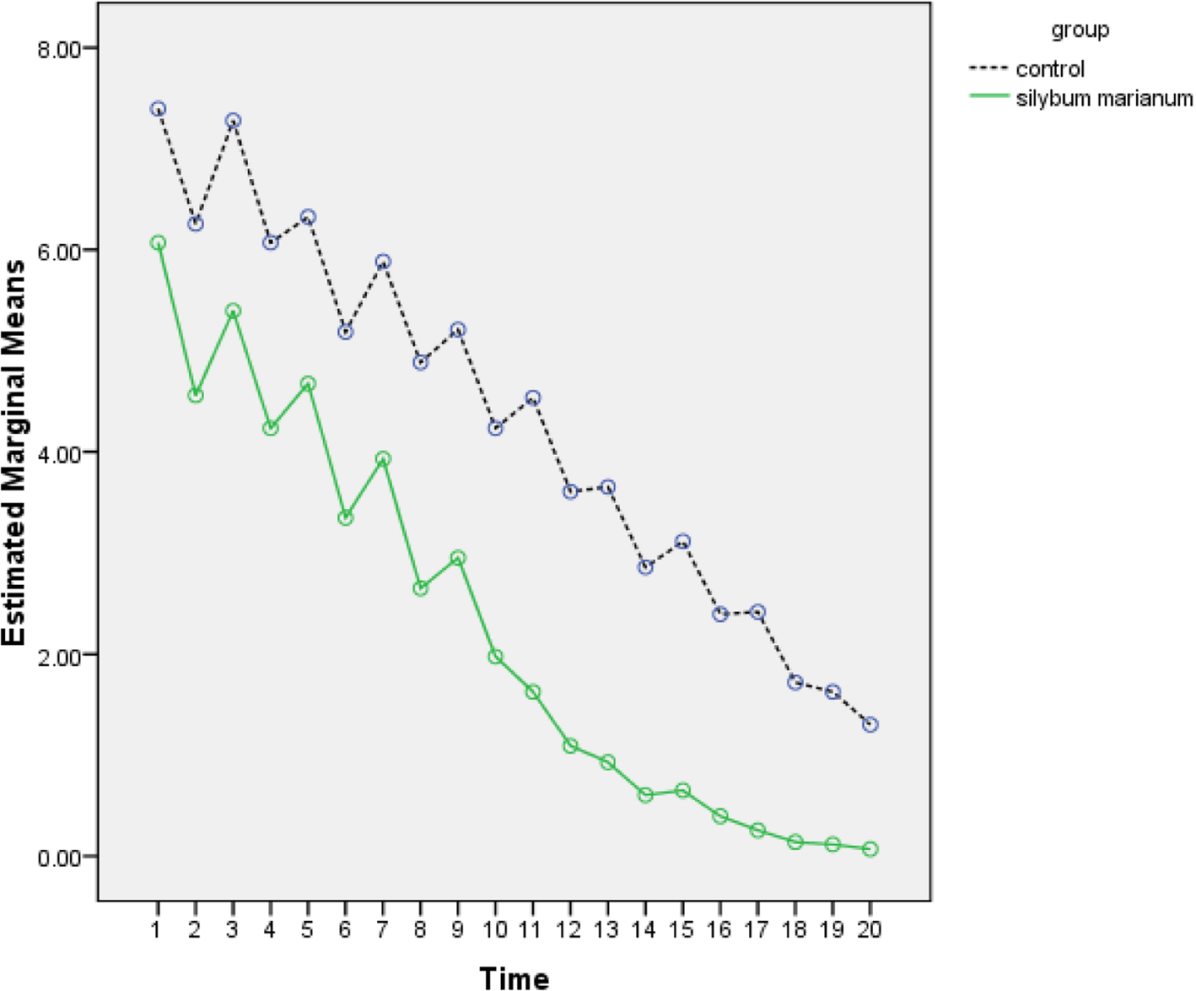
Comparison of pain severity in two groups of *S. marianum* ointment and Placebo ointment in primiparous women

Figure 3 shows pain severity in two groups at different times.

Pain severity in *S. marianum* group comparing to placebo is estimated with confidence interval of 95% in Table 3 (Table 4).

**Table 4:** Pain severity in two groups of *S. marianum* ointment and Placebo ointment with confidence interval of 95%

Parameter	Estimate	STD.error	*p*-value	95% Confidence Interval	
				Lower Bound	Upper bound
group	−0.973	0.184	.000	−1.339	−0.981

No serious side effect for the drug was observed in the study except for itching and burning which was the same in two groups of *S. marianum* ointment and placebo ointment (one case of itching and dryness and two cases of topical burning in intervention group, and 4 cases of topical skin dryness in control group at fifth day of using). Thereby, it was recommended to use washing serum until compete relief of the problem and there was no more need to an additional treatment.

## Discussion

In the current study, *S. marianum* ointment was effective in healing wound and decline in severity of episiotomy pain comparing to placebo ointment group.

Only one study has been done to investigate *S. marianum* orally on wound recovery on human samples to date [42], and this is the first time that the efficacy of topical form of this herb is thoroughly investigated in a controlled clinical trial on wound healing and pain decline in humans.

Results of a study which investigated the effect of *S. marianum* on wound healing in rats showed that topical use of *S. marianum* leads to wound recovery regarding lesser redness, exudates and swelling. The researchers found that *S. marianum* ointment is a promising therapeutic agent to treat wounds through antioxida*nt* and anti-inflammatory property [40].

In the present study also at fifth and tenth day after labor, redness and edema in *S. marianum* group comparing to placebo group is significantly lower which shows lesser inflammation in *S. marianum* group.

The result of a study accomplished on skin wounds induced in mice by the *Leishmania major* showed that, sylimarin gel in both concentration of 5 and 10% accelerate wound closure and also improve collagen synthesis and vascular regeneration through increase in length density, volume density and mean diameter of blood vessels [54].

In addition, the pain mean score in the intervention group showed significant statistical difference comparing to the control group. In an experimental study sedative effects of sylimarin which was used intra-peritoneally and its interaction with histamine H1 receptors in 42 wistar rats was assessed and analgesic effects of sylimarin was investigated using Formalin test by emphasizing histaminergic neurotransmitter system in wistar rats. The results showed that sylimarin has analgesic effects which probably are induced through histamine inhibition [56].

The study by Jadhav, G. B et al. conducted on mice showed that sylimarin herbal medicine has analgesic property which probably is induces through inhibition of prostaglandin synthesis [57]. Flavonoids (including silymarin) are good antioxidants [58], it seems that this compound implements its pain decreasing effects through inhibition of releasing harmful enzymes and histamins which causes allergy and swelling. In addition, flavonoids can control pain centrally through various mechanisms including affecting on gama amino butiric acid (GABA)receptors and inhibiting enzymes involved in brain [59]. The effect of flavonoids on pain reduction has been confirmed in a similar study which aimed to examine the effect of combinatory ointment of chamomile and calendula on episiotomy pain severity [60].

This study despite attempts in minimzing probable errors had limitations, including lack of possibility of controlling factors such as indivdiual differences of samples regarding perinea tissue, nutritional status and physical movement in each indivdiual which was tried to control it by providing identical trainings and random allocations.

In addition, since perception of pain and expression of it is different in various individuals, it might approximately affect the results, however it was tried to decrease distribution of pain severity measurement among samples using a standard ruler of pain record, blinding and random allocation.

The strengths of the study were triple masking, controlling entry of confounding variables at the beginning of sampling, following up the individuals by phone, and assessment of wound healing by two independent researchers.

## Conclusion

*S. marianum* is effective in accelerating wound healing process and decreasing episiotomy pain without inducing any complication and the participants experienced lesser pain and discomfort after labor. The current study can be an instruction on starting studies related to *Silybum marianum* in future specifically for wound healing. Although, our clinical trial has been conducted on a small sample and highly selected primiparous populations, this does not mean that these findings are not generalizable to the general community of pregnant women with episiotomy, but rather that the beneficial effects of s.marianum should be assessed on individuals with the least main inclusion criteria like older mothers and who differ in terms of their baseline characteristics from participants recruited in this study. Moreover, it is recommended to conduct further studies regarding efficacy of various doses and probable side effects of *S. marianum* on wound healing.

## Additional file

**Additional file 1.** CONSORT 2010 checklist of information to include when reporting a randomised trial*.

## Data Availability

The datasets used and/or analyzed during the current study are available from the corresponding author on reasonable request.

## Abbreviations

s.marianum: *Silybum marianum*
REEDA Scale: Redness(R), Edema (E), Ecchymosis (E), Discharge from the wound (D), Approximation of the perineal tissues
IRCT: Iranian Registry of Clinical Trials
